# Association between neutrophil-to-high-density lipoprotein cholesterol ratio (NHR) and psoriasis risk: a nationally representative cross-sectional study

**DOI:** 10.3389/fimmu.2025.1611867

**Published:** 2025-08-19

**Authors:** Huazheng Liang, Wenyue Si, Huilan Huang, Lin Li, Xin Li, Kaiying Yang

**Affiliations:** ^1^ Department of Pediatric Surgery, Guangzhou Women and Children’s Medical Center, National Children’s Medical Center for South Central Region, Guangzhou Medical University, Guangzhou, China; ^2^ School of Pediatrics, Guangzhou Medical University, Guangzhou, China; ^3^ Department of Science Research and Education Management, Guangzhou Women and Children’s Medical Center, National Children’s Medical Center for South Central Region, Guangzhou Medical University, Guangzhou, China; ^4^ The Second School of Clinical Medicine, Guangzhou Medical University, Guangzhou, China; ^5^ Department of Pediatric Surgery, Guangdong Provincial Key Laboratory of Research in Structural Birth Defect Disease, Guangzhou Women and Children’s Medical Center, Guangzhou Medical University, Guangdong Provincial Clinical Research Center for Child Health, Guangzhou, China; ^6^ Laboratory of Clinical Proteomics and Metabolomics, Institutes for Systems Genetics, Frontiers Science Center for Disease-related Molecular Network, West China Hospital, Sichuan University, Chengdu, China

**Keywords:** psoriasis, NHR, high-density lipoprotein cholesterol, cross-sectional study, national health and nutrition examination survey

## Abstract

**Objective:**

To investigate the association between the neutrophil-to-high-density lipoprotein cholesterol ratio (NHR) and the risk of psoriasis.

**Methods:**

This cross-sectional study analyzed data from the National Health and Nutrition Examination Survey (NHANES) for the periods 2003–2006 and 2009–2014, including 21,723 adults aged ≥20 years. Weighted multivariable logistic regression models were used to examine the association between NHR and psoriasis, with stepwise adjustments for demographic, metabolic parameters, and comorbid factors. Subgroup analyses, sensitivity analyses and smoothed curve fitting were conducted to assess the robustness and potential nonlinearity of the association.

**Results:**

The prevalence of psoriasis was 2.75%. Multivariable regression revealed a significant positive association between elevated NHR levels and psoriasis risk (unadjusted model: OR = 1.11, 95% CI = 1.06–1.16, *p* < 0.001; fully adjusted model: OR = 1.08, 95% CI = 1.02–1.14, *p* = 0.007). Participants in the highest NHR quartile (Q4) exhibited a 63% higher risk of psoriasis compared to those in the lowest quartile Q1 (OR = 1.63, 95% CI = 1.27–2.08, *p* < 0.001). Subgroup analyses demonstrated consistent associations across most strata, although the relationship was significantly modified by alcohol consumption history (*p* for interaction = 0.048). The sensitivity analyses substantiate NHR as a temporally stable and confounder-independent biomarker for psoriasis risk, as evidenced by consistent effect estimates across multiple analytical models and population strata.

**Conclusion:**

Higher NHR levels are independently associated with an increased risk of psoriasis. Further prospective cohort studies and mechanistic experiments are needed to validate its predictive performance and potential role in psoriasis risk stratification and monitoring.

## 1 Introduction

Psoriasis is a chronic immune-mediated inflammatory dermatosis affecting approximately 2-3% of the global population (1–3). Clinically, it is characterized by well-demarcated erythematosquamous plaques, typically distributed symmetrically on extensor surfaces (elbows, knees), scalp, and lumbosacral regions, though extensive cutaneous involvement may occur (4, 5). Beyond its cutaneous manifestations, psoriasis exerts a substantial impact on a patient’s quality of life and imposes significant socioeconomic burdens (6). Despite considerable advances in therapeutic options, post-treatment relapse rates exceed 90% (7, 8), and the patients frequently develop comorbid arthritis, metabolic syndrome, and cardiovascular complications (9–11). Current understanding of psoriasis pathogenesis implicates multifaceted interactions between immune dysregulation, genetic susceptibility, autoantigens presentation, and environmental triggers (12, 13). However, the precise molecular and cellular mechanisms remain remains incompletely understood. While biological therapies have revolutionized management, approximately 40% of patients continue to exhibit suboptimal or non-durable responses, highlighting an unmet need for clinically actionable biomarkers to facilitate personalized therapeutic strategies (14, 15).

The neutrophil-to-high-density lipoprotein cholesterol ratio (NHR) has emerged as a novel dual-functional inflammatory-metabolic biomarker, offering unique advantages by concurrently assessing innate immune activation through neutrophils and lipid metabolic disorders. Although previous studies have independently confirmed the association of neutrophils (key inflammatory mediators) and high-density lipoprotein (HDL) cholesterol (a regulator of lipid metabolism and inflammatory) with psoriasis, the combined predictive value of these two components has not been thoroughly investigated (16, 17). Mechanistically, neutrophil extracellular traps (NETs) containing LL-37 and RNA components can directly contribute to the formation of psoriatic plaques (20). HDL typically exerts anti-inflammatory effects by modulating macrophages activity (21). However, in patients with psoriasis, HDL displays pro-inflammatory changes and impaired cholesterol efflux capacity (22). These findings suggest a dual disturbance in inflammation and metabolism that may synergistically exacerbate the development of psoriasis. Moreover, recent studies have shown that NHR demonstrates superior predictive performance compared to other inflammatory-metabolic indicators such as the monocyte-to-HDL ratio (MHR) and neutrophil-to-lymphocyte ratio (NLR), emphasizing its potential clinical utility (23).

Although NHR has shown prognostic utility in cardiovascular and metabolic diseases, its role in immune-mediated inflammatory diseases, particularly psoriasis, remains underexplored (18–20). This cross-sectional study, based on the National Health and Nutrition Examination Survey (NHANES) database, is the first to systematically evaluate the association between NHR and psoriasis. The findings offer new insights into the interplay between inflammation and metabolism in the pathogenesis of psoriasis and support the potential of NHR as a novel biomarker for clinical risk stratification and individualized therapeutic strategies.

## 2 Materials and methods

### 2.1 Data source and study participants

The NHANES, conducted by the Centers for Disease Control and Prevention (CDC), is a nationally representative, cross-sectional survey that employs a complex multistage probability sampling design to collect comprehensive health and nutritional data from the non-institutionalized U.S. population. The dataset supports a wide range of scientific research and informs evidence-based public health policies (21). The NHANES protocol received ethical approval from the National Center for Health Statistics Ethics Review Board, and all participants provided written informed consent (22). Detailed study protocols and publicly accessible datasets are accessible at https://www.cdc.gov/nchs/nhanes/.

**Figure 1:**
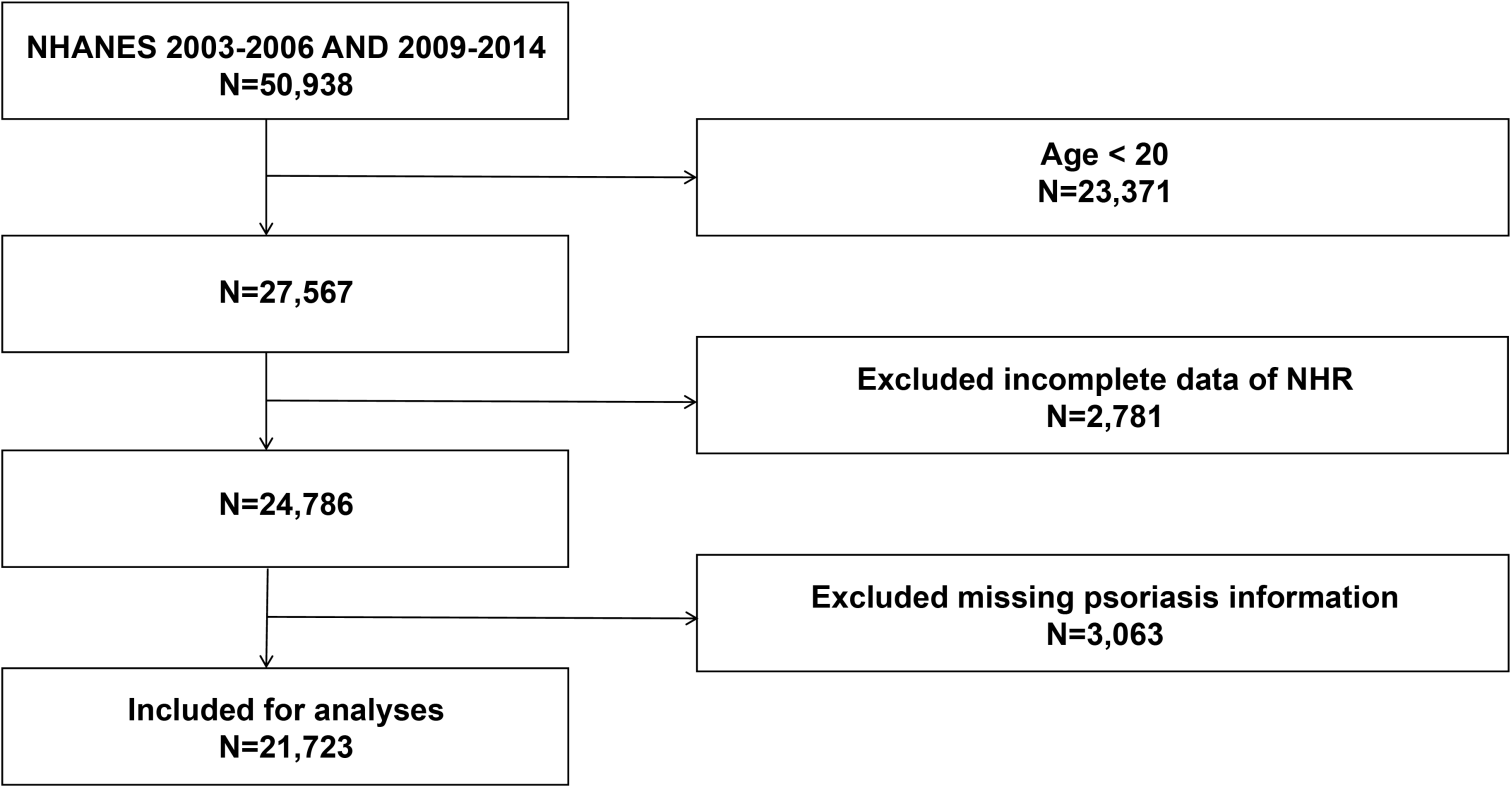
Flow chart of participants selection.

This cross-sectional study utilized data from five survey cycles (2003–2006 and 2009–2014) of the NHANES. A total of 50,938 participants were initially included. After excluding individuals younger than 20 years (n = 23,371), those with missing NHR data (n = 2,781), and those lacking incomplete information on psoriasis status (n = 3,063), the final analytical sample comprised 21,723 participants (**Figure 1**).

### 2.2 Assessment of NHR and psoriasis

Whole blood cell counts were measured using the Beckman Coulter MAXM automated hematology analyzer (Beckman Coulter Inc., USA). High-density lipoprotein cholesterol (HDL-C) concentrations were determined using a specific endpoint reaction method, with absorbance read at 600 nm to ensure measurement accuracy. Lymphocyte, neutrophil, monocyte, and platelet counts were reported as ×10³ cells/μL. The NHR was calculated by dividing the absolute neutrophil count (×10³ cells/μL) by HDL-C concentration (mmol/L) (23).

Psoriasis diagnosis was based on affirmative responses to either of the following NHANES survey questions: (a) *“Has a healthcare provider ever diagnosed you with psoriasis?”* or (b) *“Has a doctor or other health professional ever told you that you have psoriasis with eye involvement?”* (24). Participants who refused to answer or responded “don’t know” were excluded according to predefined criteria (25).

### 2.3 Covariates

Potential confounders of the NHR-psoriasis association were systematically extracted from NHANES demographic, physical examination, questionnaire, and laboratory datasets. Continuous variables included age (years), HDL-C concentration (mmol/L), and lymphocyte/neutrophil/monocyte/platelet counts (×10³ cells/μL). Categorical variables encompassed sex (male/female); race/ethnicity (Mexican American, Other Hispanic, Non-Hispanic White, Non-Hispanic Black, Multiracial/Other); education level (less than high school, high school/equivalent, and above high school); and marital status (married/cohabiting, widowed/divorced/separated, never married). Medical history variables (diabetes, hypertension, cancer, and cardiovascular disease) were included as binary indicators (yes/no). Socioeconomic status was assessed using the family income–poverty ratio (PIR), categorized as low (<1.3), middle (1.3–3.5), and high (>3.5). Body mass index (BMI) was stratified as underweight/normal (<25 kg/m²), overweight (25–29.9 kg/m²), and obese (≥30 kg/m²). Alcohol consumption was defined as non-drinker (<12 drinks/year) or drinker (≥12 drinks/year), and smoking status as non-smoker (lifetime <100 cigarettes) or smoker (≥100 cigarettes).

### 2.4 Statistical analysis

Data analyses were conducted using EmpowerStats statistical software (version 2.0; X&Y Solutions Inc., Boston, MA, USA; www.empowerstats.com). Continuous variables were expressed as weighted means ± standard errors, while categorical variables were presented as weighted frequencies (percentages). Participants were stratified by psoriasis status, and group differences were assessed using χ² tests (categorical variables) or Kruskal-Wallis tests (non-normally distributed continuous variables). Weighted multivariable logistic regression models were employed to evaluate the association between NHR and psoriasis, with odds ratios (ORs) and 95% confidence intervals (CIs) calculated across three models: Model I (unadjusted), Model II (adjusted for age, sex, and race/ethnicity), and Model III (fully adjusted for age, sex, race/ethnicity, BMI, smoking status, education level, marital status, alcohol intake, PIR, hypertension, cancer, diabetes, and cardiovascular disease). Subsequent analyses examined the relationship between NHR quartiles and psoriasis risk. We employed Generalized Additive Models (GAMs) with smoothing splines to examine the non-linear relationship between NHR and psoriasis risk. To evaluate the potential influence of clinical confounders on the study outcomes, we conducted interaction analyses. For assessing interaction terms, we followed established statistical guidelines by including multiplicative interaction terms in the models, and considered a two-tailed p-value <0.05 as statistically significant. Moreover, to comprehensively assess the robustness of the association between NHR and psoriasis risk, we performed systematic sensitivity analyses using multiple approaches. These include a primary analysis in the full population, subgroup validation after excluding cancer patients, and stratified analyses across different NHANES survey cycles (2003–2006 and 2009-2014).

## 3 Results

### 3.1 Characteristics of the study population

A total of 21,723 participants were included in the study, with a mean age of 46.02 years. The cohort predominantly comprised Non-Hispanic Black individuals, and 10,467 (48.18%) were male. The overall prevalence of psoriasis was 2.75%, with affected individuals exhibiting a higher mean age (49.27 ± 16.20 years). Clinical characteristics stratified by psoriasis status are summarized in **Table 1**. Statistically significant differences (all *p* < 0.05) were observed in age, race/ethnicity, education level, marital status, PIR, BMI, hypertension, diabetes, cancer, smoking, cardiovascular disease, HDL-C levels, lymphocyte/monocyte/neutrophil counts, as well as NHR. Compared to individuals without psoriatic, those with psoriasis tended to be older and exhibited higher NHR levels. The prevalence of psoriasis was higher among non-Hispanic Black participants, individuals with education beyond high school, unmarried status, BMI ≥ 25 kg/m², smokers, alcohol consumers, and those with diabetes, hypertension, cancer, or cardiovascular disease.

**Table 1:** The baseline characteristics of participants from the NHANES 2003–2006 and 2009–2014.

Characteristic	Total N = 21,723 (100%)	Non-psoriasis N =21,125 (97.25%)	Psoriasis N =598 (2.75%)	P Value
Age (year)	21,723 (100%)	45.93 ± 16.96	49.27 ± 16.20	<0.001^b^
Sex, n (%)				0.499^a^
Male	10,467 (48.18%)	10,187 (48.22%)	280 (46.82%)	
Female	11,256 (51.82%)	10,938 (51.78%)	318 (53.18%)	
Race, n (%)				<0.001^a^
Mexican American	3,502 (16.12%)	3,453 (16.35%)	49 (8.19%)	
Non-Hispanic White	1,770 (8.15%)	1,724 (8.16%)	46 (7.69%)	
Non-Hispanic Black	9,714 (44.72%)	9,347 (44.25%)	367 (61.37%)	
Other Hispanic	4,573 (21.05%)	4,498 (21.29%)	75 (12.54%)	
Other race/multiracial	2,164 (9.96%)	2,103 (9.96%)	61 (10.20%)	
Education, n (%)				0.033^a^
Under high school	5,257 (24.2%)	5,137 (24.32%)	120 (20.07%)	
High School or equivalent	4,927 (22.68%)	4,794 (22.69%)	133 (22.24%)	
Above high school	11,539 (53.12%)	11,194 (52.99%)	345 (57.69%)	
Marital status, n (%)				0.010^a^
Married/cohabiting	4,313 (19.85%)	4,216 (19.96%)	97 (16.22%)	
Widowed/divorced/separated	13,09 (60.28%)	12,737 (60.29%)	358 (59.87%)	
Never married	4,315 (19.86%)	4,172 (19.75%)	143 (23.91%)	
PIR (%)				0.032^a^
<1.30	6,527 (30.05%)	6,333 (29.98%)	194 (32.44%)	
1.30∼3.49	8,790 (40.46%)	8,579 (40.61%)	211 (35.28%)	
≥3.50	6,406 (29.49%)	6,213 (29.41%)	193 (32.27%)	
Had at least 12 alcohol drinks one year, n (%)				0.405^a^
Yes	16,359 (75.31%)	15,900 (75.27%)	459 (76.76%)	
No	5,364 (24.69%)	5,225 (24.73%)	139 (23.24%)	
Cigarette Use, n (%)				<0.001^a^
Smoked ≥ 100 cigarettes in life	9,689 (44.6%)	9,355 (44.28%)	334 (55.85%)	
Smoked < 100 cigarettes in life	12,034 (55.4%)	11,770 (55.72%)	264 (44.15%)	
Diabetes, n (%)				<0.001^a^
Yes	2,699 (12.42%)	2,591 (12.27%)	108 (18.06%)	
No	19,024 (87.58%)	18,534 (87.73%)	490 (81.94%)	
Hypertension, n (%)				<0.001^a^
Yes	6,951 (32%)	6,699 (31.71%)	252 (42.14%)	
No	14,772 (68%)	14,426 (68.29%)	346 (57.86%)	
Cancer, n (%)				<0.001^a^
Yes	1,678 (7.72%)	1,599 (7.57%)	79 (13.21%)	
No	20,045 (92.28%)	19,526 (92.43%)	519 (86.79%)	
BMI (%)				<0.001^a^
<25	6,520 (30.01%)	6,386 (30.23%)	134 (22.41%)	
25-29.9	7,325 (33.72%)	7,110 (33.66%)	215 (35.95%)	
≥30	7,878 (36.27%)	7,629 (36.11%)	249 (41.64%)	
Heart attack				<0.001^a^
Yes	732 (3.37%)	692 (3.28%)	40 (6.69%)	
No	20,991 (96.63%)	20,433 (96.72%)	558 (93.31%)	
HDL-C (mmol/L)	21,723 (100%)	1.38 ± 0.41	1.33 ± 0.42	0.001^b^
Lymphocyte number (1000 cells/uL)	21,723 (100%)	2.15 ± 0.96	2.04 ± 0.67	0.003^b^
Monocyte number (1000 cells/uL)	21,723 (100%)	0.54 ± 0.20	0.57 ± 0.18	<0.001^b^
Neutrophils count (1000 cell/uL)	21,723 (100%)	4.33 ± 1.90	4.53 ± 1.70	<0.001^b^
Platelet count (1000 cells/uL)	21,723 (100%)	248.36 ± 66.82	246.70 ± 67.07	0.662^b^
NHR	21,723 (100%)	3.40 ± 1.64	3.71 ± 1.73	<0.001^b^

Continuous variables are presented as the means ± standard deviations. Categorical variables are presented as n (%). ^a^Chi-square test, ^b^Kruskal–Wallis rank sum test. BMI, body mass index; NHR, neutrophil-to-high-density lipoprotein cholesterol ratio; PIR, family income–poverty ratio; HDL-C, High-density lipoprotein cholesterol.

### 3.2 Association between NHR and psoriasis

**Figure 2:**
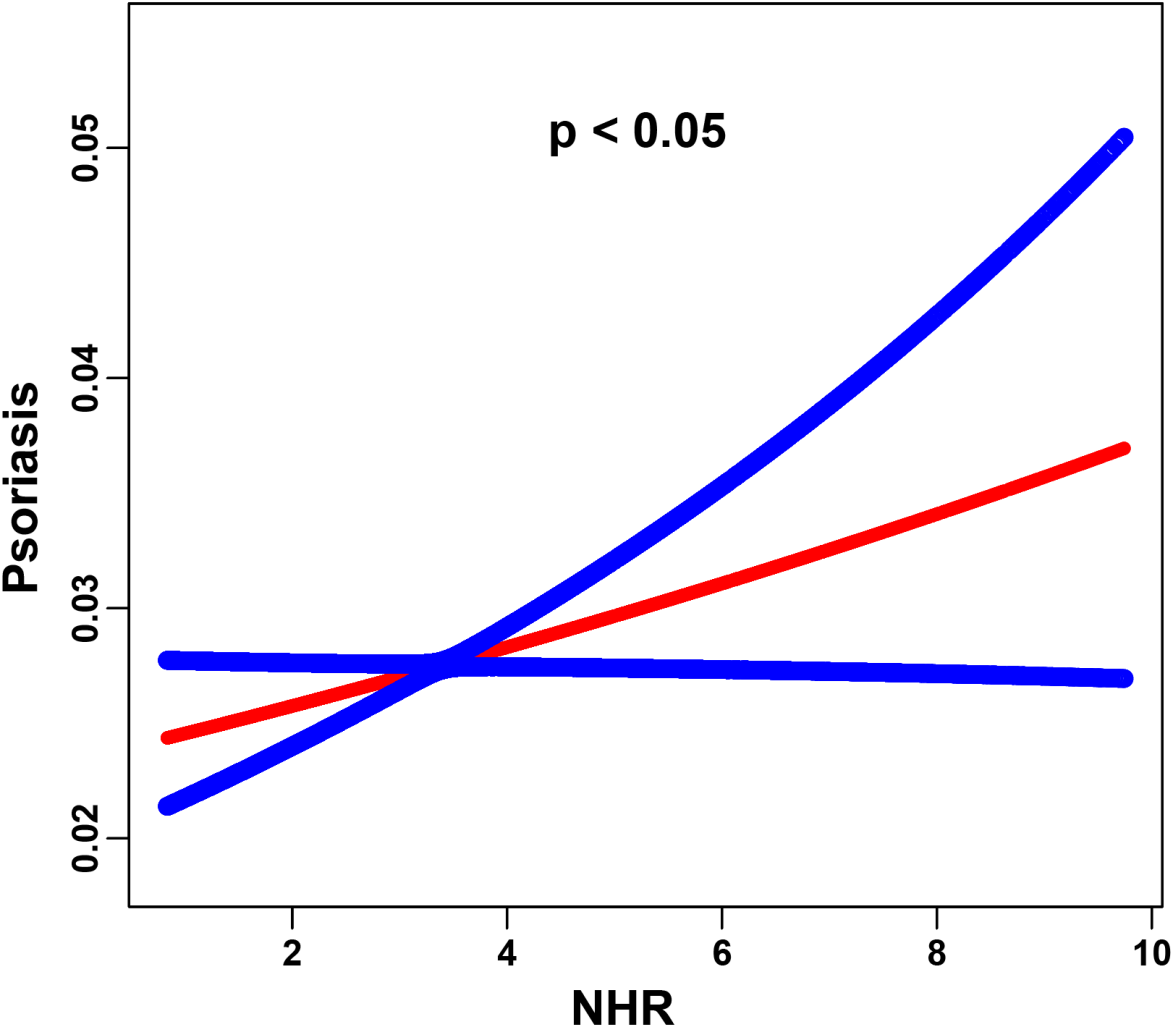
The association between the NHR and psoriasis is illustrated via smoothed curve fitting methods. Adjusted for age, sex, race, PIR, BMI, cigarette use, hypertension, education, diabetes, cancer, marital status, heart attack and alcohol use. The solid red line depicts the smooth curve fit between the variables, whereas the blue bands represent the 95% confidence intervals derived from the fit. The y-axis indicates the predicted probability of psoriasis occurrence, estimated using a logistic regression model with smoothing splines.

Stepwise weighted multivariable logistic regression models revealed a significant positive association between elevated NHR levels and the risk of psoriasis (**Table 2**). In the unadjusted model, each unit increase in NHR was associated with an 11% higher odds of psoriasis (OR = 1.11, 95% CI = 1.06-1.16, *p* < 0.001), which remained consistent after the partially adjusted model for confounders (OR = 1.11, 95% CI = 1.05-1.16, *p* < 0.001). When NHR was analyzed as a categorical variable, participants in the highest quartile (Q4) demonstrated a 63% increased risk compared to those in the lowest quartile Q1 (OR = 1.63, 95% CI = 1.27-2.08, *p* < 0.001). We further investigated the nonlinear association between the NHR and psoriasis risk using smoothing curve fitting, which revealed a statistically significant nonlinear trend (*P* < 0.05, based on GAMs). As shown in **Figure 2**, the fitted curve displayed a clear positive nonlinear correlation.

**Table 2:** Weighted associations between NHR (continuous and quartile-based) and psoriasis risk.

	OR (95% CI)		
	Model 1	Model 2	Model 3
	(N= 21,723)	(N= 21,723)	(N= 21,723)
NHR	1.09 (1.08, 1.10) <0.001	1.09 (1.08, 1.10) <0.001	1.04 (1.02, 1.05) <0.001
Stratified by NHR quartiles			
Q1	Reference	Reference	Reference
Q2	1.08 (1.02, 1.15) 0.009	1.07 (1.01, 1.14) 0.017	0.99 (0.93, 1.05)0.710
Q3	1.31 (1.24, 1.39) <0.001	1.31 (1.25, 1.40) <0.001	1.14 (1.07, 1.21) <0.001
Q4	1.52 (1.44, 1.60) <0.001	1.56 (1.47, 1.65) <0.001	1.24 (1.17, 1.32) <0.001
P for trend	<0.001	<0.001	<0.001

Model 1: adjusted for none.

Model 2: adjusted for age (years), sex, and race.

Model 3: adjusted for age (years), sex, race, PIR, BMI, cigarette use, hypertension, education, diabetes, cancer, marital status, heart attack and alcohol use.

BMI, body mass index; PIR, family income–poverty ratio; OR, odds ratio; NHR, neutrophil-to-high-density lipoprotein cholesterol ratio; CI, confidence interval.

### 3.3 Subgroup analysis

Subgroup analyses were conducted to evaluate the consistency of the nonlinear association between NHR and psoriasis across different population strata (**Table 3**). Interaction tests indicated stable associations in most subgroups, including sex, age, race/ethnicity, education level, smoking status, BMI, marital status, cardiovascular disease, PIR, hypertension, cancer, and diabetes (all *p* for interaction > 0.05). However, a significant interaction was observed for alcohol consumption history (*p* for interaction = 0.048), indicating a stronger association in individuals who did not consume alcohol.

**Table 3:** Subgroup analysis of the effect of the NHR on psoriasis (N = 21,723).

Subgroup	N	OR (95% CI)	P for interaction
Age (year)			0.852
20∼39	8,651	1.06 (0.97, 1.16)	
40∼59	7,982	1.03 (0.95, 1.11)	
≥60	5,090	1.06 (0.96, 1.17)	
Sex			0.884
Male	10,467	1.04 (0.97, 1.12)	
Female	11,256	1.05 (0.98, 1.13)	
Race			0.416
Mexican American	3,502	0.96 (0.79, 1.16)	
Non-Hispanic White	1,770	1.07 (0.89, 1.29)	
Non-Hispanic Black	9,714	1.02 (0.96, 1.09)	
Other Hispanic	4,573	1.13 (0.99, 1.31)	
Other race/multiracial	2,164	1.15 (0.98, 1.35)	
Education			0.396
Under high school	5,257	0.98 (0.87, 1.09)	
High School or equivalent	4,927	1.07 (0.96, 1.18)	
Above high school	11,539	1.06 (0.99, 1.14)	
Marital status			0.357
Married/cohabiting	4,313	1.04 (0.91, 1.18)	
Widowed/divorced/separated	13,095	1.08 (1.01, 1.15)	
Never married	4,315	0.98 (0.88, 1.10)	
BMI			0.903
<25	6,520	1.02 (0.91, 1.16)	
25∼29.9	7,325	1.05 (0.96, 1.14)	
≥30	7,878	1.06 (0.98, 1.14)	
PIR			0.649
<1.30	6,527	1.06 (0.98, 1.16)	
1.30∼3.49	8,790	1.06 (0.98, 1.15)	
≥3.50	6,406	1.01 (0.91, 1.11)	
Cigarette Use			0.625
Smoked ≥ 100 cigarettes in life	9,689	1.04 (0.97, 1.10)	
Smoked < 100 cigarettes in life	12,034	1.06 (0.98, 1.15)	
Diabetes			0.787
Yes	2,699	1.03 (0.92, 1.16)	
No	19,024	1.05 (0.99, 1.11)	
Hypertension			0.993
Yes	6,951	1.05 (0.97, 1.13)	
No	14,772	1.05 (0.98, 1.12)	
Cancer			0.760
Yes	1,678	1.07 (0.94, 1.21)	
No	20,045	1.04 (0.99, 1.10)	
Had at least 12 alcohol drinks one year			0.048
Yes	16,359	1.02 (0.96, 1.08)	
No	5,364	1.14 (1.04, 1.26)	
Heart attack			0.339
Yes	732	0.96 (0.80, 1.16)	
No	20,991	1.06 (1.00, 1.11)	

Age, sex, race, PIR, BMI, cigarette use, hypertension, education, diabetes, cancer, marital status, heart attack and alcohol use were adjusted.

BMI, body mass index; PIR, family income–poverty ratio; NHR, neutrophil-to-high-density lipoprotein cholesterol ratio.

### 3.4 Sensitivity analysis

Furthermore, we conducted comprehensive sensitivity analyses to rigorously validate the robustness of the association between NHR and psoriasis risk (**Table 4**). The fully adjusted model (Model 2) showed that each unit increase in NHR was significantly associated with a higher risk of psoriasis in the total population (OR=1.04, 95% CI: 1.02-1.05). This association remained consistent in the cancer-free subgroup (OR=1.03, 95% CI: 1.01-1.04) and in the 2009–2014 survey cycle (OR=1.06, 95% CI: 1.04-1.08), but was attenuated in the 2003–2006 cycle (OR=1.01, 95% CI: 0.99-1.03). The small change in effect size (ΔOR=0.05) between the crude and fully adjusted models suggest that the association was stable after adjusting for potential confounders, including demographic, socioeconomic, and metabolic factors.

**Table 4:** Sensitivity test.

Mode	Total	Non-Cancer	03–06 cycle	09–14 cycle
Crude	1.09 (1.08,1.10)	1.08 (1.07,1.09)	1.07 (1.05,1.09)	1.10 (1.09,1.12)
Model 1	1.09 (1.08,1.10)	1.08 (1.07,1.09)	1.07 (1.06,1.09)	1.11 (1.09,1.12)
Model 2	1.04 (1.02,1.05)	1.03 (1.01,1.04)	1.01 (0.99,1.03)	1.06 (1.04,1.08)

Age, sex, race, PIR, BMI, cigarette use, hypertension, education, diabetes, cancer, marital status, heart attack and alcohol use were adjusted. BMI, body mass index; PIR, family income–poverty ratio; NHR, neutrophil-to-high-density lipoprotein cholesterol ratio.

## 4 Discussion

To the best of our knowledge, this is the first cross-sectional study to investigate the association between NHR and psoriasis using data from the NHANES database. Weighted multivariable logistic regression analyses of 21,723 participants identified an independent positive association between NHR and psoriasis risk, consistent across both unadjusted and partially adjusted models. Subgroup analyses further confirmed the robustness of this association across various demographic and clinical subpopulations, with alcohol consumption identified as a potential effect modifier. The sensitivity analyses demonstrated that the NHR possesses both temporal stability and is robust to confounding, supporting its utility as a biomarker for psoriasis risk. These findings provide novel insights into the inflammatory-metabolic interplay underlying psoriasis and underscore the potential utility of NHR as a biomarker for risk stratification and early intervention.

Recent advancements have highlighted the clinical relevance of novel biomarkers in disease prediction and management. As a composite inflammatory-metabolic marker, NHR uniquely integrates peripheral neutrophil counts and HDL-C levels, providing a multidimensional perspective on the interplay between inflammation and lipid metabolism (26, 27). Notably, NHR has demonstrated superior predictive performance over monocyte-to-HDL ratio and neutrophil-to-lymphocyte ratio across a range of pathological conditions (18, 28). For example, Tian et al. (2025) identified elevated NHR as a predictor of aortic dissection and aneurysm risk, implicating vascular inflammatory pathways (29). Similarly, NHR was associated with disease severity in patients with acute biliary pancreatitis (30), while Du et al. (2025) reported a 34% increased risk of renal stone in individuals within the highest NHR tertile (95% CI: 1.15–1.57, *p* < 0.001) (31). Collectively, these findings support the role of elevated NHR as a marker of both neutrophil-mediated inflammation and HDL-C dysfunction, consistent with our observed association between NHR and psoriasis risk.

The observed dose-response relationship between NHR and psoriasis risk, although not yet fully elucidated mechanistically, is supported by established biological frameworks. Neutrophils play a central role in psoriasis pathogenesis and contribute to disease progression through multiple pathways: (1) activated neutrophils secrete cytokines within IL-23/IL-17 axis, promoting Th17 cell differentiation and keratinocyte hyperproliferation (32); (2) neutrophil extracellular traps, composed of DNA-LL37 complexes and histones, activate plasmacytoid dendritic cells via Toll-like receptor 9 signaling, leading to increased production of TNF-α/IL-6 release, and further activation of keratinocytes and T-cells (33, 34); (3) NADPH oxidase 2-dependent reactive oxygen species induces keratinocyte necroptosis, thereby compromising epidermal barrier integrity (35). Simultaneously, HDL in patients with psoriasis demonstrates pro-inflammatory properties and reduced cholesterol efflux capacity (36). Moreover, prospective data from the UK Biobank indicate an inverse association between HDL-C levels and psoriasis risk (37). Collectively, these mechanisms support the “neutrophil activation–HDL dysfunction” axis as a biologically plausible pathway underlying the association between NHR and psoriatic pathology.

Furthermore, the study found that elevated NHR was more strongly associated with psoriasis risk among participants who did not consume alcohol. This observation may be explained by the absence the dual protective effects associated with low-to-moderate alcohol intake in this group. First, low-dose ethanol has been shown to exert anti-inflammatory effects via the gut microbiota-acetate-GPR43 axis. This pathway inhibits neutrophil reactive oxygen species production and endoplasmic reticulum stress, thereby reducing the formation of NET (38). Second, moderate alcohol consumption has been reported to increase HDL-C levels by enhancing the transport of apolipoprotein A-I and A-II, which improve HDL function (39). Together, these mechanisms suggest that low-to-moderate alcohol consumption may help alleviate psoriasis-related inflammation and metabolic disturbances by reducing NET formation and enhancing HDL function. However, it is important to note that excessive alcohol intake may negate these potential benefits, and that individual responses vary significantly (40, 41).

The characteristics of the study population may have influenced the observed associations. In this study, the term “drinkers” referred to individuals who consumed at least 12 alcoholic beverages per year. This category includes participants with very low levels of alcohol intake, which may attenuate the potential protective effects of low-to-moderate alcohol consumption. Considering these factors, elevated NHR was found to be significantly and strongly associated with psoriasis risk in the non-drinking group.

This study has several strengths. First, it is the first systematic evaluation of NHR in the context of chronic inflammatory skin disease. Second, the use of a nationally representative NHANES cohort enhances the generalizability of the findings. Third, rigorous multivariable adjustments were employed to minimize potential confounding, and stratified analyses were conducted to account for population heterogeneity. However, several limitations should be acknowledged. First, the cross-sectional design limits the ability to draw causal inferences. Second, psoriasis diagnoses were based on self-reported reports, which may induce potential misclassification bias. Future prospective cohort studies and mechanistic experiments are needed to validate the predictive utility of NHR and to elucidate its molecular effects on keratinocyte biology. Moreover, as the current analysis was restricted to U.S. adult males, future multicenter studies across diverse healthcare systems and ethnic populations are necessary to confirm the generalizability of NHR as a biomarker for psoriasis.

Clinically, the accessibility and low cost of NHR make it a practical tool for psoriasis risk assessment. Early interventions targeting metabolic-inflammatory pathways in individuals with elevated NHR may help prevent or slow disease progression. Moreover, longitudinal monitoring of NHR could enhance the prediction of therapeutic response to biologics, thereby contributing to the advancement of personalized management in psoriasis.

## 5 Conclusion

This study reveals a significant positive association between the NHR and psoriasis risk. Our findings provide translational evidence supporting early risk stratification and precision interventions in psoriasis, highlighting the role of inflammatory-metabolic interplay. Future prospective cohort studies involving larger and more diverse populations are needed to confirm this relationship and further elucidate its underlying mechanisms.

## Data Availability

The datasets presented in this study can be found in online repositories. The names of the repository/repositories and accession number(s) can be found below: https://www.cdc.gov/nchs/nhanes/.
